# Topical Use of Quercetin-Loaded Chitosan Nanoparticles Against Ultraviolet B Radiation

**DOI:** 10.3389/fphar.2018.00826

**Published:** 2018-07-26

**Authors:** Wenhao Nan, Li Ding, Houjie Chen, Fahim U. Khan, Lu Yu, Xinbing Sui, Xiaojun Shi

**Affiliations:** ^1^School of Life Sciences, Tsinghua University, Beijing, China; ^2^Graduate School at Shenzhen, Tsinghua University, Shenzhen, China; ^3^Shenzhen Modo Biotech Co., Ltd., Shenzhen, China; ^4^Department of Medical Oncology, Holistic Integrative Oncology Institutes and Holistic Integrative Cancer Center of Traditional Chinese and Western Medicine, The Affiliated Hospital of Hangzhou Normal University, College of Medicine, Hangzhou Normal University, Hangzhou, China; ^5^Department of Cancer Pharmacology, Holistic Integrative Pharmacy Institutes, College of Medicine, Hangzhou Normal University, Hangzhou, China

**Keywords:** Quercetin, chitosan, nanoparticles, topical use, anti-UVB radiation, skin damaging

## Abstract

Ultraviolet radiation is a major risk factor for human skin damage, especially solar ultraviolet-B (UVB) which can induce inflammation, photoaging, and skin cancer. Quercetin (Qu), one of flavonoid family members, has showed protective effects against UVB radiation. However, its application for topical use is limited by low hydrophilicity and poor percutaneous absorption. Herein, we found that Qu, if entrapped into TPP-Chitosan nanoparticles (TCs), can be efficiently uptake by HaCaT cells and easily permeate through the epidermis layer, meanwhile display better stability and low cytotoxicity. We also found that Qu-loaded TCs (QTCs) could notably enhance the effect of Qu on inhibiting the NF-kB/COX-2 signaling pathway as well as ameliorating the skin edema caused by UVB radiation. Therefore, this study provided a method to get rid of Qu’s low hydrophilicity, enhance its percutaneous absorption and retention in the skin, and further improve its anti-UVB effect, and demonstrated that Qu-loaded chitosan nanoparticles can be used as the therapeutic agent for topical use against UVB radiation.

## Introduction

Previous studies have reported that the ultraviolet radiation (UVR) harms the human skin, particularly in fair skin populations (Madronich and Gruijl, 1993; Gallagher and Lee, 2006; Morganroth et al., 2013). Currently, due to gradual depletion of the ozone layer caused by chemical compounds containing gaseous chlorine or bromine from industries or human in atmosphere, the solar ultraviolet-B (UVB; 280–315 nm) radiation significantly penetrates to Earth’s surface resulting in skin diseases including erythema, sunburn, inflammation, photoaging, oxidative stress, DNA damage, immunosuppression, and even skin cancer (Kerr and Mcelroy, 1993; Pal et al., 2015).

One of the most important mechanisms involved in UVB radiation caused skin damage is the activation of NF-κB/cyclooxygenase-2 (COX-2) signaling pathway. COX-2 one of the enzyme plays a key role in converting arachidonic acid (AA) into prostaglandins (PGE2) which increases the vascular permeability and promotes edema (Kim et al., 2005; Liu et al., 2009; Hur et al., 2010; Pal et al., 2015; Tang et al., 2017). In addition, high COX-2 level are frequently correlated with skin inflammation and cancer development (Pentland et al., 1999; Tang et al., 2008; Mccarty, 2012; Jiao et al., 2014). Once our skin suffers from UVB radiation, IkB-α is phosphorylated through a family of serine/threonine kinases known as IKK, and subsequently falls off from NF-κB protein complex, which drives NF-κB translocating from the cytoplasm to the nucleus. Furthermore, this kind of NF-κB translocation enhances the expression of COX-2 (Kim et al., 2005; Liu et al., 2009; Hur et al., 2010; Pal et al., 2015; Tang et al., 2017).

Many flavonoids including Quercetin (Qu) play a vital role in regulating NF-κB/COX-2 signaling pathway (Serafini et al., 2010; de Alencar Filho et al., 2016). Qu is one representative flavonoid with anti-oxidant, anti-inflammation, and anti-tumor properties (Formica and Regelson, 1995). Previous research has revealed that Qu is very effective against UVB radiation through downregulating COX-2 level *in vitro* (Steerenberg et al., 1997). However, *in vivo* application of Qu is limited by its low hydrophilicity and poor percutaneous absorption (Hung et al., 2012). Therefore, many researchers managed to seek for new dosage forms of Qu to solve this problem, including Qu-loaded liposome or nanoparticles (NPs) (Hatahet et al., 2016). Our previous study has also reported using PLGA-TPGS NPs to load Qu (Zhu et al., 2016). However, although the application of PLGA-TPGS NPs significantly enhanced the protective effect of Qu, we think it is still not ideal preparation of Qu considering the natural disadvantages of PLGA-TPGS NPs. That is, PLGA-TPGS NPs, owing negative surface charge, will no doubt impede the nano-bio interaction with skin cell cytomembrane which also negatively charged (Yang et al., 2009).

Chitosan (CS), an abundant linear cationic biopolymer, has several favorable biological characteristics such as biodegradability, non-toxicity, biocompatibility, and anti-pathogen (Enríquez et al., 2006). Most importantly, CS NPs (CSs) can increase the cellular uptake due to their positive surface charge which can enhance the interaction with negatively surface charged cytomembrane (Schipper et al., 1997; Huang et al., 2002). Another advantage of CSs is its enhancement of the skin permeation of hydrophobic drugs and promotion of the retention of drugs in the epidermis, due to its interaction with the skin surface which will change the morphology of the stratum corneum and break the close conjugation of the corneocyte layers (Tan et al., 2011). Thus, theoretically Qu, if loaded into CSs, may efficiently overcome the shortcomings of Qu for topical use to protect skin from UVB radiation damage.

In this work, we compared the protective effects of Qu and Qu-loaded TPP-Chitosan NPs (QTCs) against UVB radiation *in vitro* and *in vivo*, using HaCaT cells and C57BL/6 mice models. We found that QTCs which displayed better stability and low cytotoxicity could be uptake by HaCaT cells efficiently and easily permeate through stratum corneum and epidermis. Besides, the application of CSs carrier enhanced the effect of Qu on inhibiting the NF-κB/COX-2 signaling pathway, further ameliorating the skin edema induced by UVB radiation. In this study, we firstly showed the advantages of Qu-loaded CS NPs for topical use against UVB radiation-induced skin damage.

## Materials and Methods

### Chemicals

Quercetin and coumarin-6 were purchased from Sigma-Aldrich (St. Louis, MO, United States). Chitosan (deacetylation degree ≥ 95%, viscosity 100–200 mpa.s, biotechnology level) and sodium tripolyphosphate (TPP) were purchased from Macklin (Shanghai, China). 4’,6-Diamidino-2-phenylindole dihydrochloride (DAPI) and dimethyl sulfoxide (DMSO) were purchased from Sangon Biotech (Shanghai, China). Wheat Germ Agglutinin Alexa Fluor 594 conjugate (WGA-594) were purchased from Thermo Fisher Scientific (Eugene, OR, United States). All other chemicals and reagents of the highest quality were commercially available and used as received. Antibodies against p-IkB-α, NF-κB, COX-2, GAPDH, β-actin and lamin A/C were purchased from Cell Signaling Technology.

### Formulation and Characterization of NPs

The preparation of TPP-Chitosan NPs (TCs) was based on ionic gelation of chitosan with TPP anions reported before (Zhang et al., 2008), but with some modifications. Under magnetic stirring at room temperature, 20 mg CS was dissolved into 10 ml 1% (V/V) acetum and adjusted the pH to 5.5, then 3.5 ml TPP with the concentration of 2 mg/ml was dropwise added into CS solution. Opalescent and transparent solution was formed after transient sonication. Qu loaded TPP-Chitosan NPs (QTCs) were formed by dropwise adding 1 ml Qu ethanol solution with the concentration of 3 mg/ml into CS solution before adding TPP. The NPs were centrifuged at 8,000 rpm, 4°C for 30 min, the supernatant was collected for HPLC and the precipitation was suspended after washed three times by ddH_2_O, and finally lyophilized.

The hydrodynamic diameter and zeta potential of NPs were measured by Dynamic Light Scattering Zetasizer (Zetasizer Nano ZS90, Malvern Instruments Ltd., Malvern, United Kingdom). The morphology of NPs was showed by transmission electron microscopy (TEM, Tecnai G20, FEI Company, Hillsboro, OR, United States).

### Measurement of Drug Encapsulation Efficiency and Drug Loading Content

The drug encapsulation efficiency (EE) and drug loading content (LC) of the QTCs were measured by HPLC (LC 1200, Agilent Technologies, Santa Clara, CA, United States). The mobile phase of HPLC was 0.2% phosphoric acid solution and MeOH with the volume ratio of 55:45. The flow rate of mobile phase was 1 ml/min. The UV detection wavelength was 360 nm. A C-18 column (150 mm × 4.6 mm, GL Science Inc., Tokyo, Japan) was used. The supernatant of QTCs after centrifugation was used for HPLC to detect the concentration of Qu in the supernatant; therefore, we can detect the amount of Qu in supernatant (W_1_). We named the weight of lyophilized precipitation as W_2_ and the amount of Qu added in the preparation of QTC as W_3_.

$$EE=(W_{3}-W_{1})/W_{3}\times 100\%$$

$$LC=(W_{3}-W_{1})/W_{2}\times 100\%$$

### Cell Culture

HaCaT cells were cultured in Dulbecco’s modified Eagle’s medium (DMEM; Gibco BRL, United States) supplemented with 10% fetal bovine serum (FBS, Gibco) at 37°C with 5% CO_2_.

### Cellular Uptake of Coumarin-6-Loaded TCs and Its Efficiency

Coumarin-6 was used as the fluorescent probe which was entrapped into TCs to show the cellular uptake of QTCs. HaCaT cells were seeded in 12-well plate containing cover glasses in the bottom. After the cells adhered to the glasses, 100 μg/ml coumarin-6-loaded TCs was added to the culture medium and incubated at 37°C for 3 h. Then removed the culture medium and washed with PBS three times. Next added WGA-594 diluted by PBS to the wells and incubated at 37°C for 20 min to stain the cytomembrane, then washed with PBS three times and fixed with 4% paraformaldehyde for 20 min, finally, cells were stained with DAPI for 15 min and washed with PBS three times. Thereafter, cells were investigated by confocal laser scanning microscope (CLSM, Olympus Fluoview FV-1000, Tokyo, Japan).

To quantitatively measure the cellular uptake efficiency of coumarin-6-loaded TCs, 100 μg/ml coumarin-6-loaded TCs were incubated with HaCaT cells in 12-well plate at 3, 6, 12, 24 (each group, *n* = 3), and the quantitative ratio of coumarin-6 fluorescence in cytoplasm were measured by detecting the absorbance value at 320 nm.

### Cytotoxicity and UVB Protective Experiment *in Vitro*

Cytotoxicity was evaluated by MTT assay. Cells were seeded in 96-well plate, after the cell attachment rate reached 60∼70%, replaced the culture medium to new culture medium mixed with or without different concentrations of Qu (mixed with DMSO, at the concentration of no influence on cells), QTCs, TCs and incubated for another 24 h. Then removed the culture medium, added new culture medium mixed with MTT (0.5 mg/ml) and incubated 4 h. Finally, the absorbance value of 490 nm was detected.

The method to investigate the UVB protective effects of Qu, QTCs, and TCs *in vitro* were almost the same as cytotoxicity experiment. The difference was the time of incubation with different concentrations of Qu, QTCs, and TCs been cut to 8 h, then removed the culture medium and irradiated using a microprocessor-controlled UV Crosslinker (XL-1000, SPECTROLINKER^TM^, United States) of 12 mJ/cm^2^. After the radiation, cells were added new drug free culture medium and returned to the incubator immediately.

### Immunoblotting

Cell and tissue lysates were separated by 12% SDS-PAGE and analyzed by immunoblotting using P-IkB-α, NF-κB, COX-2 antibodies, followed by enhanced chemiluminescence (ECL) detection. Isolation of nuclear protein was conducted using the manufacturer’s protocol (Abcam, Cambridge, MA, United States).

### Immunocytochemical Staining of NF-κB

Cell culture and UVB radiation were the same as the UVB radiation treatment mentioned before. 10 h after UVB radiation, the cells were fixed and immunofluorescence stained with NF-κB antibody. After stained with DAPI, the cells were detected by CLSM.

### Percutaneous Absorption and Retention Study of QTCs and Qu Aqueous Solution

All animal experiment protocols were approved by the Administrative Committee on Animal Research in the Graduate School at Shenzhen, Tsinghua University. Female C57BL/6 mice (aged 6 weeks, weighing about 20 g) were purchased from Guangdong Medical Laboratory Animal Center. After execution, the dorsal skin was carefully shaved with electric clippers and then carefully cut down the dorsal skin. Scrape the subcutaneous tissue completely and carefully by using a knife, and then fixed the skin on the V-C diffusion cell to investigate the percutaneous absorption of QTCs aqueous solution and Qu aqueous solution (each group, *n* = 3). Take samples regularly on 2, 4, 6, 8, 12, and 24 h for HPLC. The preparation of QTCs aqueous solution was mentioned before, and the preparation of Qu aqueous solution was by dropwise adding 1 ml Qu ethanol solution (5 mg/ml) to 9 ml ddH_2_O under magnetic stirring.

### Skin Permeation of QTCs and Its Mechanism

Coumarin-6-loaded TCs was applied to represent skin permeation of QTCs visually. After the mice dorsal skin was carefully shaved with electric clippers, Coumarin-6-loaded TCs aqueous solution (80 mg/20 ml) was applied every 3 h, and after 12 h the treated skin was excised for frozen section to observe the skin permeation of Coumarin-6-loaded TCs. Divided the dorsal skin shaved mice into three groups (one group was for control with no treatment) and used the same method to treat shaved dorsal skin by H_2_O and QTCs aqueous solution, then the treated skin was excised and the paraffin section was processed for hematoxylin and eosin (HE) staining to observe the difference of stratum corneum between different treated skins.

### UVB Radiation on Animal, Staining, and Histopathological Analysis

After anesthesia with chloral hydrate, the dorsal skin of C57BL/6 mice was carefully shaved with electric clippers, depilated with depilatory paste (Veet^®^) and divided into five groups (one group was for control), then ddH_2_O, 10 mg/20 ml Qu aqueous solution and 80 mg/20 ml QTCs and TCs aqueous solution were applied to the dorsal skin every 8 h. Three days later, UVB radiation was applied at the intensity of 400 mJ/cm^2^. After radiation, the four kinds of aqueous solutions were applied every 8 h. After 48 h, the mice were executed. The aqueous solution treated areas were excised, cut into small pieces, part of which were prepared paraffin sections and protein extraction. The protein extraction was used for immunoblotting, and the paraffin sections were used for HE and Masson staining which were further used for histopathological analysis. The other part of treated skin pieces was used for detecting the content of PGE2. Respectively weigh 100 mg different treated skin pieces, added into 1 ml PBS (pH 7.2) after cut into small pieces with intermittent ultrasonic for 1 h and then detected the absorbance value at 278 nm, Using the absorbance value per gram of skin (A/g) to represent the content of PGE2.

### Statistical Analysis

All data are presented as the mean ± SEM of no less than three independent experiments. Comparisons were performed using a two-tailed paired Student’s test (^∗^*P* < 0.05, ^∗∗^*P* < 0.01, ^∗∗∗^*P* < 0.001, ^∗∗∗∗^*P* < 0.0001).

## Results

### Preparation and Characterization of NPs

**FIGURE 1:**
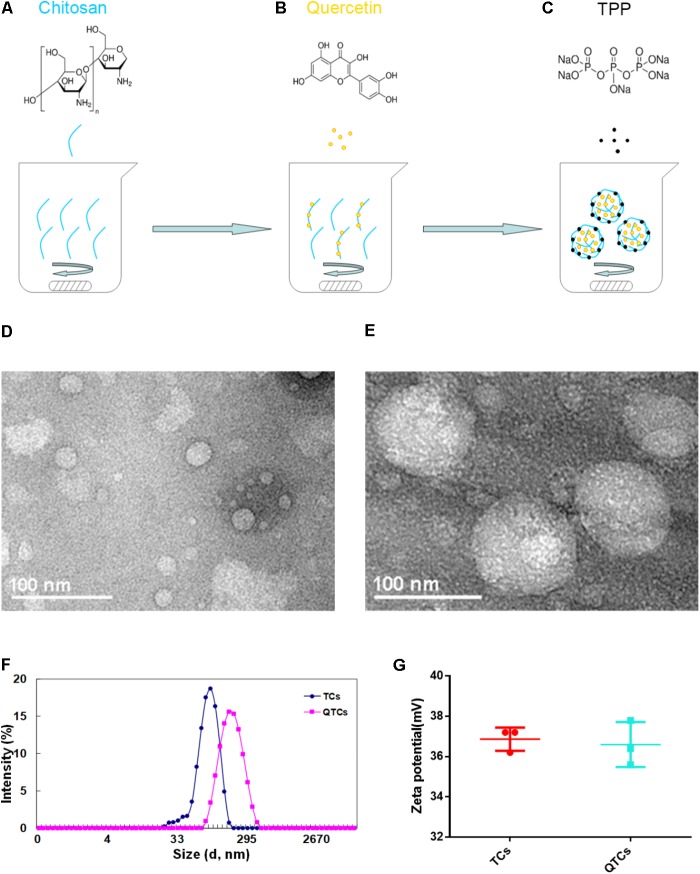
Preparation and characterization of QTCs. **(A–C)** Scheme of preparation of QTCs. **(D)** TEM image of TCs and **(E)** QTCs. **(F)** DLS size distribution of TCs and QTCs. **(G)** Zeta potential of TCs and QTCs.

We prepared the QTCs using the published protocol, but with minor modification (Zhang et al., 2008). As we presented in **Figures 1A–C**, Qu can firstly adhere to CS based on similarity-intermiscibility theory, and then formed nanospheres through TPP-CS crosslinking under continuously stirring condition. Next, the morphologies of QTCs and TCs were observed. From the TEM image, QTCs and TCs were all nearly spherical in shape, but the size between each was quite different (**Figures 1D,E**). The dynamic light scattering (DLS) assay showed that the average size of TCs was about 89.48 ± 2.03 nm while QTCs was about 183.63 ± 1.52 nm in **Figure 1F**, consistent with the results of TEM. Moreover, zeta potential, an essential index in the stability of NPs in suspension through electrostatic repulsion between the NPs, had been tested. As the result presented in **Figure 1G**, both QTCs and TCs showed almost the same zeta potential, about 37 mV in average. This data implied that both QTCs and TCs were highly stable in suspension. Then the drug EE and LC of QTCs were measured by HPLC. EE and LC of QTCs were 90.98 ± 1.66% and 13.15 ± 0.77%, respectively, indicating the excellent drug carrier ability of QTCs.

### Cellular Uptake, Cytotoxicity, and UVB Protective Effect of QTCs *in Vitro*

**FIGURE 2:**
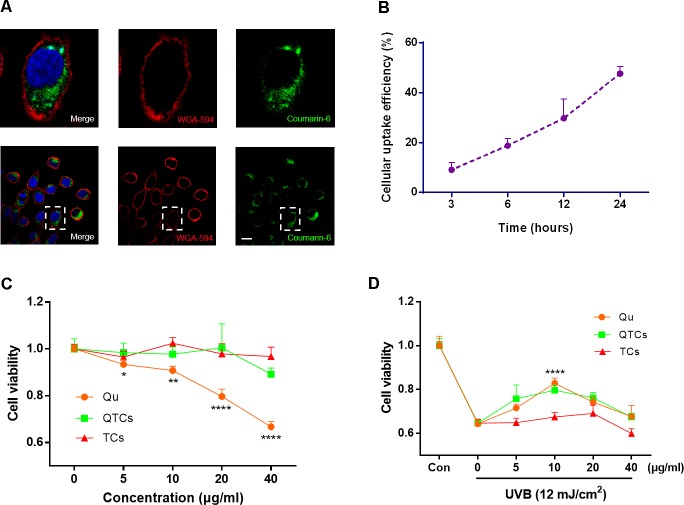
Cellular uptake, cytotoxicity, and UVB protective effect of QTCs *in vitro*. **(A)** Cellular uptake of coumarin-6-loaded TCs (100 μg/ml) was tested by CLSM after 3 h incubation with HaCaT cells. The images above are the enlarged ones in the white box on the below images. Cytomembrane was labeled with WGA-594 (red). Scale bar: 10 μm. **(B)** The cellular uptake efficiency of coumarin-6-loaded TCs (100 μg/ml) was measured at indicated times. **(C)** Cytotoxicity evaluation of Qu, QTCs, and TCs after 24 h incubation with HaCaT cells. **(D)** UVB protective effect evaluation of Qu, QTCs, and TCs. HaCaT cells were firstly incubated with Qu, QTCs or TCs, respectively, for 8 h and then treated with 12 mJ/cm^2^ UVB radiation. Concentration of QTCs was at the same drug dose compared to Qu, and that the TCs was at the same nanoparticles concentration with QTCs (^∗^*P* < 0.05, ^∗∗^*P* < 0.01, ^∗∗∗∗^*P* < 0.0001).

Many studies have been reported that CSs could enhance cellular uptake because their positive surface charge can promote the bio-nano interaction with the cytoplasmic-membrane which has negative surface charge (Schipper et al., 1997; Huang et al., 2002), which is also the reason why CSs have been often used as gene delivery carrier (Özbaş-Turan and Akbuğa, 2011). In our study, the enhancement UVB protective effect of QTCs depends on the successful internalization and sustained retention by skin cells. Therefore, we used coumarin-6 which is a kind of fluorescent dye, instead of Qu in order to visually investigate the cellular uptake of QTCs and its efficiency. HaCaT cells, the most commonly used human skin cells, were chosen as representative cells. **Figure 2A** showed the confocal images of HaCaT cells after incubation with coumarin-6-loaded TCs for 3 h. The data suggested coumarin-6-loaded TCs can be ingested by HaCaT cells and internalized into the cytoplasm. Moreover, to investigate the cellular uptake efficiency of QTCs, we measured the quantitative ratio of coumarin-6 fluorescence in cytoplasm at indicated times after incubation of coumarin-6-loaded TCs with HaCaT cells. The result showed that HaCaT cells could almost consistently uptake coumarin-6-loaded TCs in 24 h. The cellular uptake efficiency was gradually increased and reached at about 50% at 24 h (**Figure 2B**).

Next, we evaluated the cytotoxicity of Qu and QTCs in HaCaT cells. **Figure 2C** showed the *in vitro* cell viability of the drug formulated in QTCs and Qu at equivalent concentrations of 5, 10, 20, 40 μg/ml, respectively. High dose free Qu showed obvious cytotoxicity compared with QTCs at the same equivalent concentration at 24 h, which indicated that QTCs can significantly reduce cytotoxicity of high dose Qu. Additionally, the TCs did not show any obvious toxicity against HaCaT cells. After verifying the safety of QTCs, we furthermore tried to investigate the protective effect of QTCs compared to Qu on HaCaT cells under UVB radiation. We found that QTCs had same protective effects on HaCaT cells as Qu after 12 mJ/cm^2^ UVB radiation (**Figure 2D**). At the equivalent concentration of 10 μg/ml of Qu, both QTCs and Qu showed the highest protective efficiency on UVB radiation. Meanwhile, TCs showed no significant protective effect on UVB radiation.

### QTCs Enhance the Inhibition Efficacy of Qu on the NF-κB/COX-2 Signaling Pathway *in Vitro*

**FIGURE 3:**
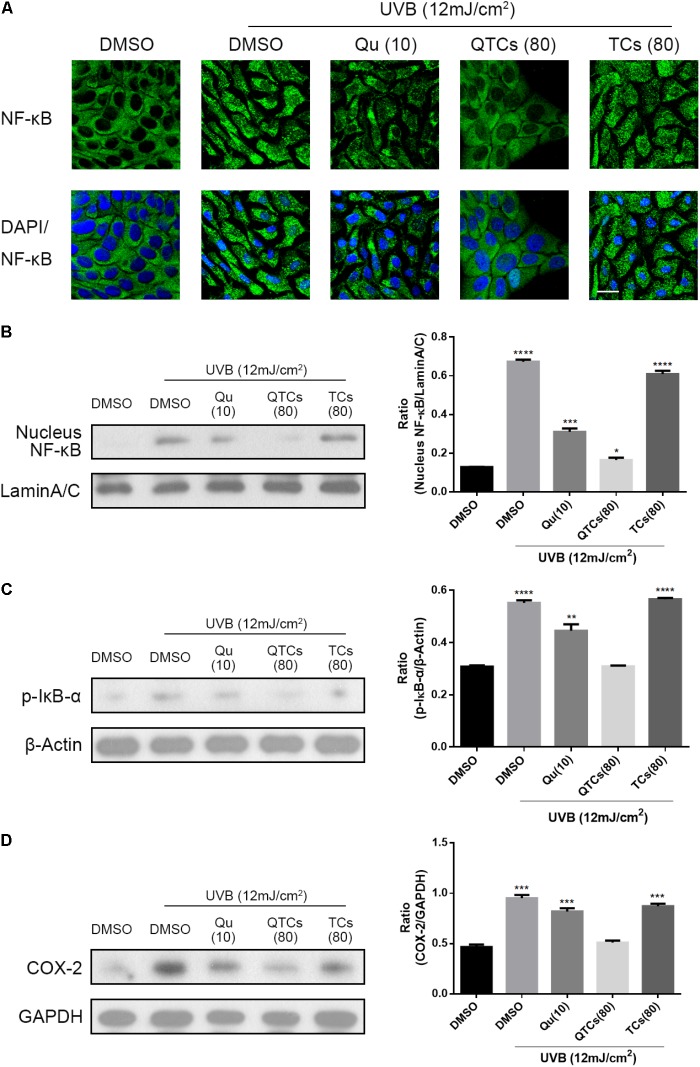
QTCs enhance the inhibition effect of Qu on the NF-κB/COX-2 signaling pathway *in vitro*. **(A)** Effect of Qu, QTCs, and TCs on the translocation of NF-κB from cytoplasm to the nuclei was visually illustrated by CLSM. Scale bar: 20 μm. **(B)** Immunoblotting analysis of the effect of Qu, QTCs, and TCs on the expression of NF-κB in nucleus. **(C)** Immunoblotting analysis of the effect of Qu, QTCs, and TCs on the phosphorylation of IkB-α. **(D)** Immunoblotting analysis of the effect of Qu, QTCs, and TCs on the expression of COX-2. Histograms represent quantitative analysis of the immunoblotting results (^∗^*P* < 0.05, ^∗∗^*P* < 0.01, ^∗∗∗^*P* < 0.001, ^∗∗∗∗^*P* < 0.0001).

Many pharmacological researches have revealed that Qu is very effective against UVB radiation *via* downregulating COX-2 level *in vitro* through NF-κB/COX-2 signaling pathway (13, 14). In non-stimulated cells, the NF-κB dimer binds to one of the three inhibitors (IkBα, IkBβ, and IkBε) and exists in an inactive state. Various signals activate NF-κB by degrading one or more of these three inhibitors (DiDonato et al., 1997). Therefore, the hallmark of NF-κB activation is that the activated NF-κB protein will enter the nucleus, and then binds to DNA in the nucleus to upregulate COX-2 level. In order to further evaluate the protective effect of QTCs, we observed immunofluorescent staining of NF-κB protein in HaCaT cells with UVB radiation in different treatment groups. As can be seen in **Figure 3A**, after 12 mJ/cm^2^ UVB radiation, NF-κB protein was translocated from the cytoplasm to the nucleus. But when pre-treatment with 10 μg/ml Qu, the NF-κB protein in the nucleus reduced. And the pre-treatment 80 μg/ml QTCs (consistent drug dose with 10 μg/ml Qu) showed more reduction compared with free Qu. TCs showed no obvious influence on this process. What must be mentioned was that the nucleus size of UVB irradiated HaCaT cells was smaller compared to normal HaCaT cells, and the morphology of UVB irradiated cells also changed to be abnormal, whereas the HaCaT cells pretreated with QTCs looked nearly normal condition after UVB irradiation. Representative western blotting image of nucleus NF-κB protein level also showed the same conclusion (**Figure 3B**). Furthermore, pre-treatment with 10 μg/ml Qu or 80 μg/ml QTCs (consistent drug dose with 10 μg/ml Qu) can also inhibit the over-phosphorylation of IkB-α caused by UVB radiation (**Figure 3C**). As a result, COX-2 protein expression level stayed relatively low in Qu or QTCs pre-treatment group (**Figure 3D**). Similarly, the effect of QTCs on these proteins was much stronger than free Qu. All of these data has proved that QTCs not only just remains the Qu-like inhibition effect on the NF-κB/COX-2 signaling pathway *in vitro*, but also enhances its therapeutic efficacy.

### QTCs Enhance the Percutaneous Absorption and Retention of Qu

**FIGURE 4:**
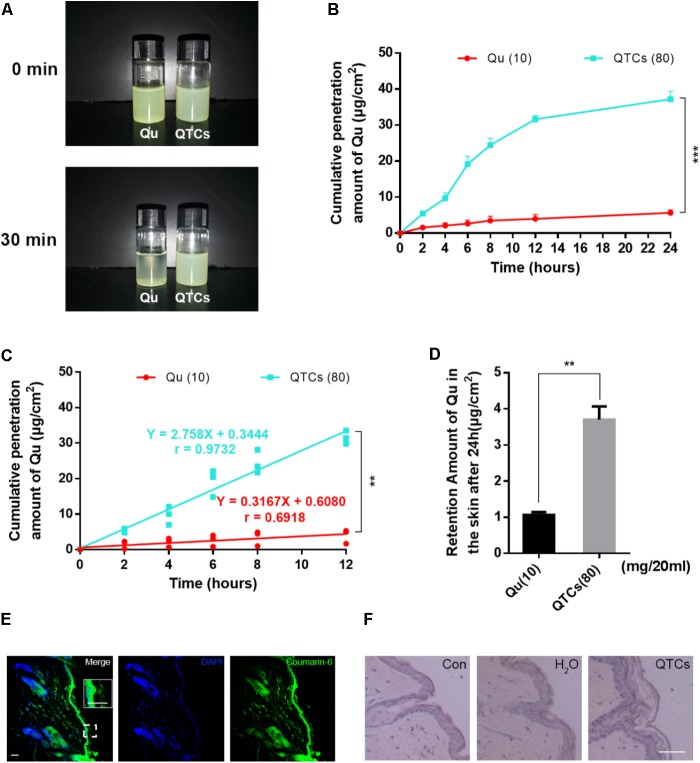
QTCs enhance the percutaneous absorption of Qu and its mechanism. **(A)** Comparison of stability between Qu aqueous solution and QTCs aqueous solution. **(B)** Cumulative penetration amount curves of Qu and QTCs in 24 h. **(C)** Cumulative penetration amount curves of Qu and QTCs in 12 h. **(D)** Retention amount of Qu in the skin after 24 h. (^∗∗^*P* < 0.01, ^∗∗∗^*P* < 0.001). **(E)** Coumarin-6-loaded TCs permeated and deposited in mice skin. The image in the white box is enlarged vision of the small white box below. **(F)** HE staining of normal, H_2_O and QTCs aqueous solution treated mice skin. Scale bars: 50 μm.

The efficiency of an external dosage form mostly depends on the percutaneous absorption. As we mentioned before, CSs is reported as nanomaterials with the advantages of enhancement of the skin permeation of hydrophobic drugs and promotion of the retention of drugs in the epidermis, due to its interaction with the skin surface will change the morphology of the stratum corneum and break the close conjugation of the corneocyte layers (Tan et al., 2011). Therefore, we next checked the contribution of CSs on percutaneous absorption and retention of Qu *in vitro*. Firstly, we conducted the percutaneous absorption experiment of QTCs and Qu and compared the efficiency of percutaneous absorption in different groups. The results shown in **Figure 4A** indicated that Qu aqueous solution was very unstable and would precipitate within 30 min because of the low hydrophilicity of Qu. And the cumulative penetration amount of Qu in aqueous solution was very small even after 24 h (**Figure 4B**). On the contrary, QTCs showed better stability in aqueous solution (**Figure 4A**) and the cumulative penetration amount of Qu in QTCs shown higher level after 24 h (**Figure 4B**). Moreover, the cumulative penetration amount of Qu in QTCs presented an upward tendency in a linear line within 12 h, as showed in **Figure 4C** the *r*-value was 0.9732 which approaches to 1, demonstrating that QTCs can significantly improve the percutaneous absorption efficiency of Qu *in vitro*. Further result shown QTCs helped the retention of Qu in skin after 24 h (**Figure 4D**).

Then, to visually show the skin permeation of QTCs *in vivo*, we also used coumarin-6-loaded TCs to represent QTCs. As showed in **Figure 4E**, after application of coumarin-6-loaded TCs aqueous solution on the shaved mice skin after 12 h, we found the coumarin-6-loaded TCs permeated and stored in the skin. Furthermore, we compared the HE staining of normal, ddH_2_O treated and QTCs aqueous solution treated mice skin, and the results showed in **Figure 4F**. The stratum corneum of QTCs aqueous solution treated mice skin was more swelling and had larger pore space than normal and ddH_2_O treated skin. This result was consistent with the enlarged vision in the white box in **Figure 4E**, which also showed that the stratum corneum of coumarin-6-loaded TCs aqueous solution treated skin was swelling. These results proved that QTCs can also significantly improve the percutaneous absorption efficiency of Qu *in vivo*.

### *In Vivo* Study of the Protective Effects of QTCs on UVB-Induced Skin Damage

**FIGURE 5:**
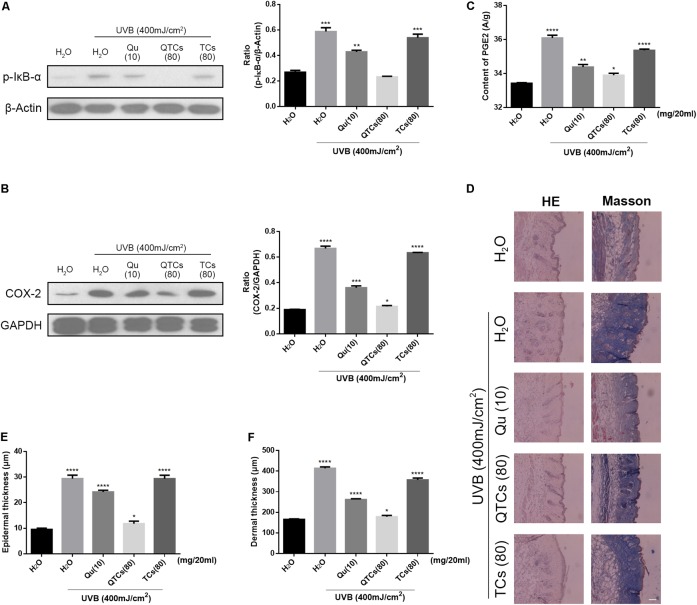
QTCs enhance the protective effect of Qu on UVB induced skin damages *in vivo*. **(A)** Immunoblotting analysis of the effect of Qu, QTCs, and TCs on the phosphorylation of IkB-α and **(B)** COX-2. Histograms represent quantitative analysis of the immunoblotting results. **(C)** The content of PGE2 in the mice skin under different treatments. **(D)** HE and Masson staining represents the alteration of epidermis and dermis, especially the thickness. Scale bar: 50 μm. **(E)** The epidermal thickness of differently treated mice. **(F)** The dermal thickness of differently treated mice. (^∗^*P* < 0.05, ^∗∗^*P* < 0.01, ^∗∗∗^*P* < 0.001, ^∗∗∗∗^*P* < 0.0001).

We further investigated the protective effects of QTCs *in vivo* after UVB radiation. We compared the effects of four different aqueous solution containing ddH_2_O, 10 mg/20 ml Qu, 80 mg/20 ml QTCs, and 80 mg/20 ml TCs in mice after UVB radiation. The immunoblotting results were shown in **Figures 5A,B**. From the results, it was obvious that QTCs had a significant enhancement effect on inhibiting NF-κB/COX-2 signaling pathway by downregulating the phosphorylation of IkB-α and the expression of COX-2 when compared with Qu. However, TCs had no notably influence on NF-κB/COX-2 signaling pathway after UVB radiation.

Moreover, PGE2 can be significantly increased after UVB radiation due to the activation of NF-kB/COX-2 signaling pathway, which can induce the increase of the vascular permeability and promotes skin edema (Kim et al., 2005; Liu et al., 2009; Hur et al., 2010; Pal et al., 2015; Tang et al., 2017). Herein, we quantificationally compared the content of PGE2 in different treated skins. Mice skin treated with QTCs showed less amount of PGE2 (**Figure 5C**). And the HE and Masson staining results in **Figure 5D**, which could help to intuitively see the alteration of mice skin after UVB radiation, also suggested the slighter disorder of collagen fiber and edema of the epidermis and dermis in mice skin treated with QTCs. The thickness of epidermis and dermis of mice also varied from different treatment. Consistent with the previous results, the status of QTCs-treated mice was most close to the normal mice skin (**Figures 5E,F**). However, TCs also had no significant difference compared with non-external preparation treatment group. With all these results, we can conclude that QTCs significantly enhanced the effect of Qu on the inhibition of the increase of PGE2 and inhibition of the edema of skin, which was closer to the normal skin, compared to Qu and TCs.

## Discussion

The pH of skin surface ranges from 5.4 to 5.9 (Eberleinkönig et al., 2000; Ali and Yosipovitch, 2013). Interestingly, the surface charge of CSs is positive in this pH, which could increase the interaction between CSs and negatively charged stratum corneum. The interaction between CSs and stratum corneum changes the morphology of the stratum corneum and breaks the close conjugation of the corneocyte layers, which means CSs could notably enhance the skin permeation of hydrophobic drugs (Tan et al., 2011). What’s more, due to the large quantities of amino groups on its chains, CSs exhibits a pH-sensitive behavior, so CSs can undergo volume phase transitions from swollen to collapsed states, which can significantly change the drug release capacity (Seda Tığlı Aydın and Pulat, 2012). Drug-loaded CSs demonstrate sustained release profiles, which may explain the promotion of drug’s retention in skin (Tan et al., 2011; Hu et al., 2014). These published data are consistent with our results. In this study, we indeed verified that Qu-loaded TPP-Chitosan NPs (QTCs) could enhance the skin permeation of Qu by changing the morphology of the stratum corneum and enhance the skin retention of Qu.

It is worth mentioning that CS has inherent antibacterial and antimycotic properties due to its polycationic nature, which can inhibit the growth of a variety of pathogenic microorganisms – Gram-positive and Gram-negative bacteria, yeasts and other fungi (Rabea et al., 2003; Ignatova et al., 2013). Furthermore, CSs could also inhibit the growth of various bacteria, including *E. coli*, *S. choleraesuis*, *S. typhimurium*, and *S. aureus* (Qi et al., 2004).

**FIGURE 6:**
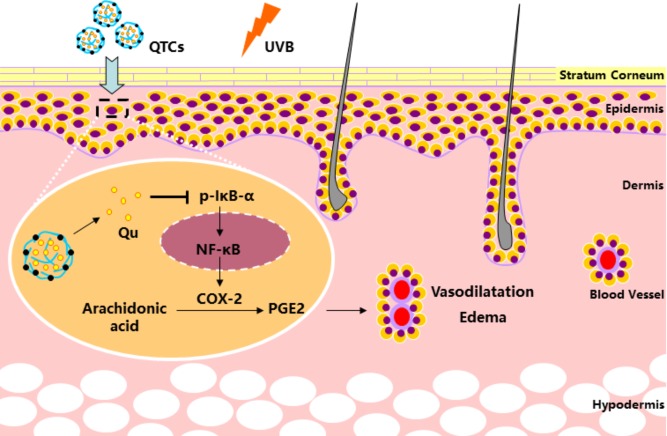
Mechanism of QTCs on enhancing the protective effect of Qu on UVB induced skin vasodilatation and edema.

Owing to these advantages, CSs can be an ideal carrier for topical drug and gene delivery (Özbaş-Turan and Akbuğa, 2011; Yang et al., 2013). Our study firstly elucidates the advantage of using Qu-loaded CSs for topical use to prevent UVB radiation-induced skin damage. As displayed in **Figure 6**, our finding showed that the UVB radiation stimulates the phosphorylation of IkB-α and subsequent degradation of IkB-α-NF-κB complex, which makes the activated NF-κB translocate to the nucleus from the cytoplasm. Nucleus NF-κB then activates the expression of COX-2, the key enzyme converting AA into prostaglandins (PGE2). Excess PGE2 increases the vascular permeability and promotes edema and further damage. Qu is well-known for inhibiting NF-κB/COX-2 pathway, but its topical use is largely restricted by its natural disadvantages of low hydrophilicity and poor percutaneous absorption. However, in QTCs drug delivery system, TCs contributes to overcome the natural disadvantages of Qu, resulting in better hydrophilicity and low cytotoxicity of QTCs drug delivery system, and also help to significantly increase the percutaneous absorption, retention and cellular uptake of Qu in the skin, which further enhances the inhibition of the NF-κB/COX-2 signaling pathway and prevents skin from UVB radiation-induced damage.

## Conclusion

Our present study firstly demonstrates that the Qu-loaded chitosan NPs have the ability to prevent the UVB radiation-induce skin damage and can be used as the promising therapeutic agent.

## Author Contributions

XjS and WN conceived the idea and designed the study. WN and LD performed all the experiments, analyzed the data, and co-wrote the paper. HC and LY provided technical support. FK helped correcting the manuscript. XjS and XbS provided reagents and conceptual advice.

## Conflict of Interest Statement

LY was employed by company Shenzhen Modo Biotech Co., Ltd. The remaining authors declare that the research was conducted in the absence of any commercial or financial relationships that could be construed as a potential conflict of interest.
